# A 2019 Social Accounting Matrix for Burkina Faso with Agricultural Activities and Household Groups Disaggregated by Agroecological Zones

**DOI:** 10.1016/j.dib.2025.112424

**Published:** 2025-12-26

**Authors:** Martial A.K. Houessou, Zuhal Elnour, Harald Grethe, François Ramdé

**Affiliations:** a Agricultural Development and Trade Group, Humboldt-Universität zu Berlin, Berlin, Germany; b Agricultural Research Corporation, Wad Madani, Sudan; c Direction des Statistiques et des Synthèses Economiques, Institut National de la Statistique et de la Démographie (INSD), Ouagadougou, Burkina Faso

**Keywords:** Social accounting matrix, Climate change, Burkina Faso, CGE model, Agroecological zones

## Abstract

A social accounting matrix (SAM) is a square matrix that captures the circular flows of all economic transactions within a country at a given time. It serves as a reference database for calibrating single-country computable general equilibrium (CGE) models to analyse the economy-wide effects of policy changes and other external shocks. We developed a SAM for Burkina Faso for 2019, adopting a top-down approach, first developing a macro-SAM and further disaggregating it to obtain a micro-SAM. The SAM depicts land as a separate account, differentiates labour by skill level, and disaggregates agriculture and households by agroecological zones. The SAM comprises 91 accounts: 47 activities, 19 commodities, margins, five factors of production, 12 household groups, three tax accounts (sales, production, and income), government, enterprises, savings-investments, and the rest of the world. The SAM is freely accessible to support economy-wide analyses for Burkina Faso, specifically, analyses across agroecological zones.

Specifications Table

**Table:** 

Subject	Social Sciences
Specific subject area	Economic Modelling, Computable General Equilibrium, Economics.
Type of data	Tables; Economy-wide database; Processed data
Data collection	The SAM development process involves various sources: 1. World Development Indicator (WDI) data 2. Integrated Regional Survey on Employment and the Informal Sector 3. 2021 National Statistical Yearbook 4. 2017 SAM for Burkina Faso 5. 2018 Harmonized Survey of Household Living Conditions (HHLC) 6. 2013 SAM for Burkina Faso 7. Unpublished 2019 SAM for Burkina Faso 8. Food and Agriculture Organization (FAO) data
Data source location	Data are collected in and on Burkina Faso.
Data accessibility	Repository name: Zenodo Data identification number: 10.5281/zenodo.14846425 Direct URL to data: https://doi.org/10.5281/zenodo.14846425
Related research article	Not applicable

## 1 Value of the Data

• The SAM differentiates agriculture activities and households by agroecological zones, which is not done in previous SAMs for Burkina Faso. It is also the first SAM with such a high level of disaggregation for households. In addition to the agroecological zones, the SAM distinguishes households by rural and urban status, as well as by income status (poor and non-poor). This results in a total of 12 household groups.
• Unlike in the existing SAMs, land is treated as a separate factor and distinguished across agricultural activities and agroecological zones, as compared to previous SAMs. This distinction improves the capacity to model land-related agricultural issues.
• The SAM provides a comprehensive level of detail relevant to simulating the economy-wide effects of policies and other external factors, allowing insights into the regional effects across agricultural subsectors and households differentiated by agroecological zones in Burkina Faso.

## 2 Background

Climate change-induced heat stress harms plants, animals, and humans globally, with the most severe effects projected in the naturally hot Sahelian regions [1]. A key consequence of these conditions is increased internal migration, as populations relocate from more severely affected areas to regions perceived as less vulnerable [1]. Burkina Faso is a Sahelian country identified as a hotspot for future climate change-induced heat stress [2] and is characterised by three distinct climatic zones.^1^ An ex-ante economy-wide analysis of climate change in such a context requires a regionalised social accounting matrix (SAM) to track human spatial mobility and depict regional effects across households and sectors. Existing SAMs for Burkina Faso (see Table S1) lack these required features. Thus, we developed a new SAM for Burkina Faso for 2019. The year 2019 is explicitly selected because it predates the short-term effects of COVID-19 and the rise in terrorist activities in recent years in Burkina Faso. This SAM incorporates several distinctive features and characteristics that distinguish it from existing SAMs for Burkina Faso.

## 3 Data Description

A social accounting matrix (SAM) is a square matrix in which each account has both a row (representing income or receipt) and a column (representing expenditure). It represents the circular flow of all transactions within an economy. As such, the total spending by each account must equal the total receipts for that account, i.e., the respective row and column sums for a SAM must be equal. Table 1 presents all SAM elements at the macro level. SAMs can be extended to include more disaggregated economic flows by adding more columns and rows. SAMs constitute the databases of computable general equilibrium (CGE) models. A SAM represents a country's economy over a given period, typically a year, and can serve as a reference to calibrate single-country CGE models for analysing the economy-wide effects of policies and other external shocks, such as climate change and fluctuations in the world market price.

**Table 1:** A social accounting matrix.

	Commodities	Activities	Factors	Households	Enterprises	Government	Investments	Rest of the world	Total
Commodities		Intermediate inputs		Household demand		Government demand	Investment demand	Commodity exports	Total demand
Activities	Domestic production								Activity income
Factors		Factor demand						Factor income from abroad	Total factor income
Households			Distributed factor income	Inter-household transfers	Distributed dividends	Transfers		Remittances	Total household income
Enterprises			Distributed factor income			Fixed (or Real) transfers		Transfers	Total enterprise income
Government	Tariff Revenue, VAT, and other taxes on commodities	Indirect taxes on activities	Distributed factor income	Direct taxes on household income	Direct taxes on enterprise income			Transfers	Total government income
Savings			Depreciation	Household savings	Enterprise savings	Internal balance		Current account balance	Total savings
Rest of the world	Commodity imports		Distributed factor income		Transfers	Transfers			Total expenditure from abroad
Total	Total supply	Gross output	Total factor expenditure	Total household expenditure	Total enterprise expenditure	Total government expenditure	Total investment expenditure	Total income from abroad	

*Source*: Adapted from Robinson and McDonald [3].

The newly developed SAM provides a snapshot of Burkina Faso’s economy as of the base year 2019. It comprises 91 accounts: 47 activities, 19 commodities, trade and transport margins, five factors of production (land, capital, unskilled, semi-skilled, and skilled labour), 12 household groups, three (sales, production, and income) tax accounts, government, enterprises, savings-investments, and the rest of the world.

The SAM saved, as “Micro SAM Balanced”, is available in the Excel file named “2019 Burkina Faso SAM.xlsx” in Zenodo (https://doi.org/10.5281/zenodo.14846425). The “2019 Burkina Faso SAM.xlsx” file encompasses three additional sheets: “Data sources,” “List of SAM accounts,” and “Macro SAM.”

**Data sources.xlsx**. The sheet summarises the sources for SAM development, as compiled in Table 2.

**Table 2:** Data sources for SAM development.

N°	Author	Title
1	World Bank [5]	World Development Indicator (WDI) data
2	INSD and AFRISTAT [4]	Integrated Regional Survey on Employment and the Informal Sector
3	INSD [9]	2021 National Statistical Yearbook
4	INSD [8]	2017 SAM for Burkina Faso
5	INSD [10]	2018 Harmonized Survey of Household Living Conditions (HHLC)
6	INSD	Unpublished 2019 SAM for Burkina Faso
7	MAHRH [7]	Unpublished 2013 SAM for Burkina Faso
8	FAO [6]	Food and Agriculture Organization (FAO) data

*Note* : **MAHRH**. Ministère de l’Agriculture, de l’Hydraulique et des Ressources Halieutiques (in French), **FAO**. Food and Agriculture Organization of the United Nations.

*Source*: Authors’ compilation.

**List of accounts.xlsx**. This sheet contains the list of 91 accounts of the balanced micro-SAM (Table S2). It also includes the standard for labour disaggregation (Table S4), the standard for agriculture (crop and livestock) disaggregation (Table S5), the standard for household disaggregation (Table S6), and a separate list of activities and commodities (Table S3).

**Macro SAM.xlsx**. The sheet contains the developed SAM, depicting Burkina Faso’s economy at a macro level, represented by column and row shares.

**Micro SAM Balanced.xlsx.** The sheet contains the developed SAM, also known as a balanced micro-SAM, depicting Burkina Faso’s economy at a micro level.

## 4 Experimental Design, Materials and Methods

**Fig. 1:**
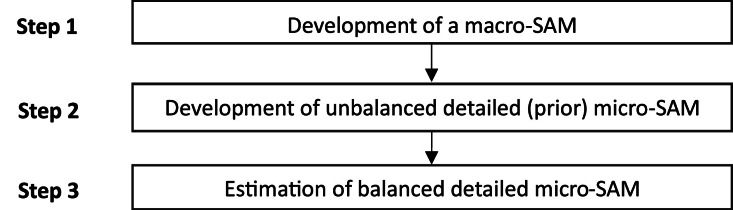
Steps in developing the 2019 SAM for Burkina Faso.

*Source*: Authors’ design.

We developed the 2019 SAM for Burkina Faso in three major steps: (1) macro-SAM development, (2) macro-SAM disaggregation into a micro-SAM, and (3) micro-SAM estimation (Fig. 1).

**Data sources.** The SAM development process involves various sources. For the macro-SAM, we mainly relied on the unpublished 2019 SAM by the National Bureau of Statistics (INSD) in Burkina Faso and the 2018 labour survey for Burkina Faso [4], while the micro-SAM development draws on a broader set of sources (Table 2).

**Development of the macro-SAM.** The macro-SAM comprises eleven accounts: commodities, activities, factors (labour and capital), institutions (households, government, and enterprises), indirect and direct taxes, savings and investments, and the rest of the world account (Table 3). It is derived from the unpublished 2019 SAM obtained from Burkina Faso’s National Bureau of Statistics (INSD), adapting factors, institutions, and the rest of the world account.

**Table 3:** 2019 macro-SAM for Burkina Faso (in billion XOF with column shares in brackets).

	Commodities (A)	Activities (B)	Labour (C)	Capital (D)	Households (E)	Government (F)	Enterprises (G)	Indirect Taxes (H)	Direct Taxes (I)	Savings & Investments (J)	Rest of the World (K)	Total (L)
Commodities (A)		BA: 5672.3 (39.6 %)			EA: 5749.2 (72.5 %)	FA: 1925.7 (94.7 %)				JA: 2117.2 (100 %)	KA: 2617.7 (78.6 %)	LA: 18,082.1
Activities (B)	AB: 14,306.9 (79.1 %)											LB: 14,306.9
Labour (C)		BC: 6379.2 (44.6 %)										LC: 6379.2
Capital (D)		BD: 2211.1 (15.5 %)										LD: 2211.1
Households (E)			CE: 6371.0 (99.9 %)	DE: 1064.6 (48.1 %)		FE: 197.0 (9.7 %)	GE: 163.4 (8.3 %)				KE: 139.3 (4.2 %)	LE: 7935.3
Government (F)								HF: 893.3 (100 %)	IF: 907.4 (100 %)		KF: 232.5 (7.0 %)	LF: 2033.2
Enterprises (G)				DG: 1146.5 (51.9 %)		FG: 112.6 (5.5 %)					KG: 701.5 (21.1 %)	LG: 1960.6
Indirect Taxes (H)	AH: 848.9 (4.7 %)	BH: 44.3 (0.3 %)										LH: 893.3
Direct Taxes (I)					EI: 334.6 (4.2 %)		GI: 572.7 (29.2 %)					LI: 907.4
Savings & Investments (J)					EJ: 1851.5 (23.3 %)	FJ: −202.2 (−9.9 %)	GJ: 1446.1 (42.3 %)				KJ: −306.6 (−10.8 %)	LJ: 2117.2
Rest of the World (K)	AK: 2926.3 (16.2 %)		CK: 8.2 (0.1 %)				GK: 395.9 (20.2 %)					LK: 3330.4
Total (L)	AL: 18,082.1	BL: 14,306.9	CL: 6379.2	DL: 2211.1	EL: 7935.3	FL: 2033.2	GL: 1960.6	HL: 893.3	IL: 907.4	JL: 2117.2	KL: 3330.4	

*Source*: Authors’ compilation.

*Commodities (column).* We retrieved the values of total commodity supply, including domestic production (cell AB), sales taxes (cell AH), and imports (cell AK) from the unpublished 2019 SAM by INSD. Domestic production, sales taxes, and imports account for 79 %, 5 %, and 16 % of total commodity supplies.

*Activities (column).* We obtained the values of domestic production, including intermediate input use (cell BA), net value-added (the sum of cells BC and BD), and production taxes (cell BH), from the unpublished 2019 SAM by the INSD. We split the net value-added into labour (cell BC) and capital (cell BD), employing shares of 74.26 % and 25.74 % computed from the 2018 labour survey [4], respectively. The production cost is composed of intermediate inputs (39.6 %), labour (44.6 %), capital (15.5 %), and production taxes (0.3 %).

*Factors—labour and capital (columns).* Labour income is distributed to domestic households (cell CE: 99.9 %) and foreign households (cell CK: 0.1 %). Foreign labour income (cell CK) is obtained from the unpublished 2019 SAM by INSD, and domestic labour income (cell CE) is the difference between total labour income (cell CL) and foreign labour income (cell CK). Shares of capital income to households (cell DE) and enterprises (cell DG) in total capital income (cell DL) represent 48.1 % and 51.9 %, respectively. Enterprises’ capital income (cell DG) is obtained from the unpublished 2019 SAM by INSD, while household capital income (cell DE) is obtained as a difference.

*Households (column).* Households spend their income on commodities (cell EA: 72.5 %), income taxes (cell EI: 4.2 %), and save the remaining (cell EJ: 23.3 %). The values of household consumption and income taxes are obtained from the unpublished 2019 SAM by INSD, and savings are calculated as residual.

*Government (column)*. Values of recurrent government expenditure (cell FA), aggregate transfers to households (cell FE), and transfers to enterprises (cell FG) are obtained from the unpublished 2019 SAM by INSD. Government savings (cell FJ) are calculated as residual.

*Enterprises (column).* Enterprise direct taxes (cell GI), capital transfers to households (cell GE), savings (cell GJ), and capital transfers to the rest of the world (cell GK) are obtained from the unpublished 2019 SAM.

*Indirect and direct taxes (column)*. Total indirect taxes (cell HF) equal the sum of sales taxes (cell AH) and production taxes (cell BH). Total direct taxes (cell IF) equal the sum of income taxes paid by households (cell EI) and enterprise direct taxes (cell GI).

*Savings and investments (column)***.** We retrieved the value of gross fixed capital formation (cell JA) from the unpublished 2019 SAM.

*Rest of the world (column)*. We obtained export values (cell KA), foreign remittances (cell KE), foreign transfers to the government (cell KF), and foreign savings (cell KJ) from the unpublished 2019 SAM. Capital transfers from the rest of the world to enterprises (cell KG) are calculated as residuals.

**Development of an unbalanced, detailed (prior) micro-SAM.** We extended macro-SAM (Table 3) into a more detailed micro-SAM, consisting of 91 accounts (Table S2 lists accounts). These include 47 activities, 19 commodities, trade and transport margins, five factors of production (land, capital, unskilled, semi-skilled, and skilled labour), 12 household groups, three (sales, production, and income) taxes, government, enterprises, savings-investments, and the rest of the world. We disaggregated indirect taxes into two, separating sales taxes from production taxes. We disaggregated the activities, labour, commodities, and household accounts.

Previous SAMs for Burkina Faso incorporate land within aggregate capital, limiting their applicability for analysing land-intensive production. In this micro-SAM, the capital account is therefore divided into separate capital and land factors for crop and forestry activities. The value of land (390 billion XOF^2^) is computed as the opportunity cost of agricultural land in 2019. We applied a 6.1 % real annual interest rate [5] of 2019 to an average land purchasing price of 500,000^3^ XOF per hectare (based on expert estimates) multiplied by the total hectares of land used in crops and forests in 2019 [6]. For agricultural activities, we deduct these land payments from the initial capital payments (1064 billion XOF), thereby reallocating returns between land and capital in a way that better reflects sectoral cost structures.

*Commodity accounts*. We first disaggregated the commodity account into five (crop, livestock, mining, manufacturing, and services), employing shares from the unpublished 2019 SAM. These comprise shares of domestic output, sales taxes, commodity imports, and commodity demand, respectively. We employed similar shares from the 2013 SAM [7] to further split crop, livestock, and manufacturing into eight, five, and two categories, respectively (Table S3 lists the commodities).

*Trade and transport margins*. We used shares from the unpublished 2019 SAM to depict the margins by commodity.

**Fig. 2:**
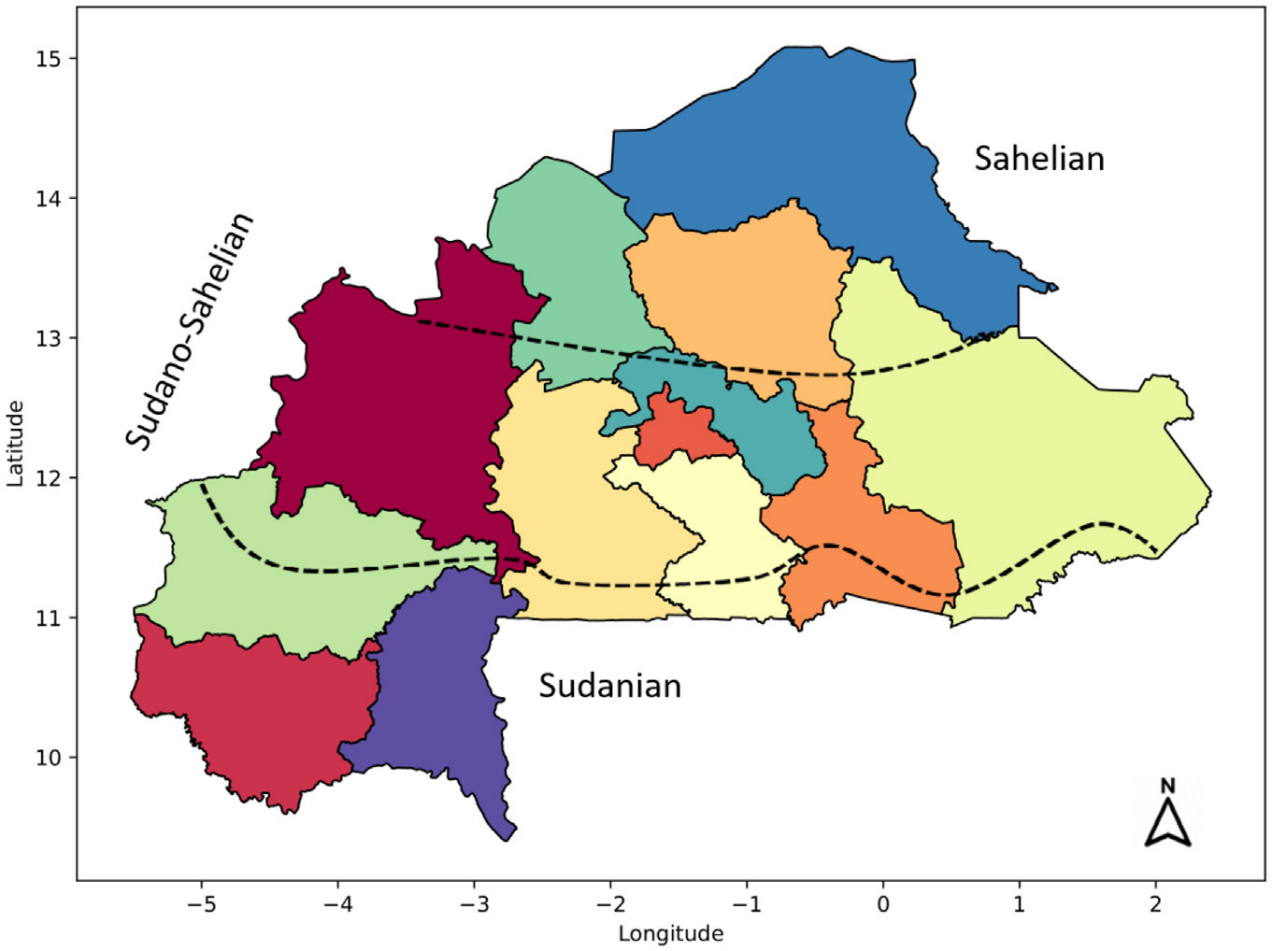
Map of Burkina Faso depicting the three agroecological zones.

*Source*: Authors’ design.

*Activity accounts.* We also disaggregated the activity account into five sectors (crop, livestock, mining, manufacturing, and services), employing shares from the 2017 SAM [8] and unpublished 2019 SAM. We used shares of sectoral value-added and production taxes from the unpublished 2019 SAM, whereas sectoral intermediate consumption shares are obtained from the 2017 SAM [8], to address inconsistencies in the unpublished 2019 SAM. Subsequently, we expanded the five aggregate sectors into 47 activities, comprising nine crop and five livestock activities produced in the three agroecological zones (Fig. 2), as well as two manufacturing, construction, mining, and service activities. The disaggregation of crop, livestock, and manufacturing activities involves two steps. First, we split crops into nine subsectors, livestock into five subsectors, and manufacturing into construction, food and non-food manufacturing activities using production cost structures (shares of intermediate inputs and production factor costs) derived from the 2013 SAM [7] for Burkina Faso. For crops and livestock, we relied on the 2019 production data to derive production outputs for the nine crops^4^ and five livestock categories.^5^ The land account is split across the nine agricultural sub-sectors using land use data from FAO [6] and INSD [9]. Second, we split each crop and livestock activity into three categories (Sudanian, Sudano-Sahelian, and Sahelian) based on 2019 production data [9] for Burkina Faso. The Sahelian zone comprises the regions a) Sahel, b) Nord, and c) Centre-Nord, the Sudano-Sahelian zone comprises a) Boucle de Mouhoun, b) Centre, c) Est, d) Centre-Est, e) Centre-Sud, f) Centre-Ouest, and g) Plateau-Central, and the Sudanian zone comprises a) Haut-Bassins, b) Cascades, and c) Sud-Ouest (Fig. 2).

By relying on the 2013 SAM for further disaggregation, we do not account for differences among changes in production cost structure across subsectors between 2013 and 2019 (see Tables S7 and S8). Compared with 2013, the share of intermediate input use has increased by 13.5 percentage points in agriculture, 4.3 percentage points in mining, and 3 percentage points in manufacturing in 2019 (see Tables S7 and S8). In agriculture, intermediate input use has primarily increased for manufacturing commodities, while in mining and manufacturing, it has increased primarily for services (see Tables S7 and S8). Conversely, the share of value added has decreased by 13, 5, and 3 percentage points in these three sectors, respectively. Likely, these changes in the cost structure of aggregate sectors are not equally distributed over subsectors, as they may have developed differently. Due to missing information, though, we assume equal changes when disaggregating subsectors based on 2013 shares. This is a potential weakness of the SAM development procedure arising from the aim of achieving a high level of disaggregation.

*Labour accounts*. Labour is disaggregated into three types: unskilled, semi-skilled, and skilled. We relied on labour revenue data (annual physical labour unit employment multiplied by the average yearly income for each labour type) by sector, based on education level, from the 2018 labour survey [4]. We categorise labour as follows: unskilled workers have no formal or primary education, semi-skilled workers have a lower secondary education level (i.e., they have completed four years of secondary school), and skilled workers have upper secondary education and above.

*Household accounts*. We disaggregated household accounts into twelve categories: rural-poor, rural-non-poor, urban-poor, and urban-non-poor, across the three agroecological zones. We followed a stepwise approach to achieve this level of disaggregation. First, we divided national household income into three groups, corresponding to the three agroecological zones, based on the 2018 household survey [10]. We further divide each of the three groups into rural and urban, also based on the 2018 household survey [10]. This results in a total of six household groups, which we further divided into poor and non-poor categories, based on the 2013 SAM [7]. Second, for expenditure, we employed shares from the 2013 SAM [7] to establish expenditure shares for rural and urban households across the three zones. Additionally, we categorised each rural and urban household group as either poor or non-poor, also based on expenditure shares from the 2013 SAM [7].

Our reliance on the 2013 SAM [7] for further household disaggregation is justified as follows. The 2018 household survey [10] used for the first-level disaggregation, lacks sufficient data to disaggregate household expenditures into twelve categories. The survey presents household expenditure shares only for food, transport, rent, durable goods, health, education, and other services. At a more disaggregated level, the survey does not provide household expenditure shares for each specific crop, livestock, and other commodities, as presented in the new SAM. Hence, reliance on the 2013 SAM [7], the most detailed SAM (see Table S1), enables us to achieve the level of detail required for the new SAM. To justify our approach, we derived aggregate household expenditure shares from the 2013 SAM [7] and benchmarked them against the 2018 household survey [10] shares (see Table S7). We constructed new household expenditure components, including food, non-food manufacturing, services, and other expenses, to make the shares from the two sources comparable. We mapped household expenditure components from each source into the four aggregate categories (see note under Table S7). We observed deviations, ranging from 0.6 to 13 percentage points. Households increased their expenditure shares on non-food manufacturing, services, and other expenses by 10, 2, and 1 percentage points, respectively, while the share of food declined by 13 percentage points in 2018 (see Table S7). At the SAM balancing stage (see below), we accounted for these deviations by setting an estimating objective that statically adjusts household consumption expenditure in the final SAM to meet the aggregate 2018 shares as closely as possible.

**Micro-SAM balancing**. The prior micro-SAM composed at step 2 is unbalanced and thus inconsistent, as total revenues (rows) and expenditures (columns) do not equate for all accounts. This is due to the reliance on different data sources. We used a Cross-Entropy (CE) approach [3], a widely used econometric optimisation technique, to estimate the prior micro-SAM under the consistency condition. The approach is Bayesian in spirit and simultaneously estimates all cells of the SAM using all available information, including prior estimates of the degree of measurement error for every cell and information about any aggregates [3]. In our case, we used the macro-SAM (Table 3) as a control to estimate the balanced micro-SAM. Specifically, we fixed all GDP components and allowed the intermediate input matrix, based on an older dataset, to adjust freely. We also used the prior micro-SAM block totals as a control. We introduced these controls to ensure that the final estimated/balanced micro-SAM is consistent with the macro-SAM and aggregate sectoral values.

Overall, the relative deviations between the unbalanced and balanced micro-SAMs range from 1 % to 30 %, in terms of total rows and columns. The most considerable deviations are present in the cells associated with activity and commodity accounts, as presented in an aggregate micro-SAM format (see Table 4). This is due to the different data sources used for disaggregating activity and commodity accounts. As explained above, these accounts were disaggregated at various levels, relying each time on a different data source. First, we used shares from the unpublished 2019 SAM to disaggregate them into five accounts each. Specifically for activity accounts, we used shares from the 2017 SAM [8] for intermediate inputs used in activities. Furthermore, we used the 2013 SAM [7] for a more detailed disaggregation into 19 accounts each. Additionally, we utilised the 2019 domestic production data [9] to disaggregate agricultural activities by agroecological zones, resulting in 47 distinct activity accounts. This process yields a significant difference between row and column totals for these accounts, which is adjusted during the SAM balancing process.

**Table 4:** Cell absolute deviations after SAM balancing/estimation (percentage change of row and column totals in brackets).

	C-Cr	C-Lv	C-Ex	C-Mf	C-Sv	A—Cr	A-Lv	A-Ex	A-Mf	A-Sv	L	K	Land	hhd	gov	entr	Itax	Dtax	sav-inv	row	total
C-Cr							8		166	0				144					32	212	568 (25 %)
C-Lv							1		258	16				−11					57	0	233 (26 %)
C-Ex								−2	−1	0									−10	−245	−258 (−13 %)
C-Mf						−34	−54	490	−552	127				−778					−79	79	−800 (−10 %)
C-Sv						0	−1	−97	−147	−202				750	0				0	−46	257 (5 %)
A-Cr	−368				0																−368 (−29 %)
A-Lv		−116			0																−116 -(21 %)
A-Ex			−16	0	0																−16 (−1 %)
A-Mf	1		0	−1231	39																−1191 (−30 %)
A-Sv	−1	0	0	−9	1699																1690 (27 %)
L						−337	−3	−369	−573	1282											0
K						−4	−84	−74	−366	528											0
Land						0															0
hhd											0	0	0		0	0				0	0
gov																	0	0		0	0
entr												0			0					0	0
Itax	0	0	0	−27	27	0	0	−1	0	0	0	0	0							0	0
Dtax											0	0	0	0		0				0	0
sav-inv														0	0	0				0	0
row	−8	0	0	−241	249						0					0					0
total	−376 (−28 %)	−115 (−21 %)	−16 (−1 %)	−1508 (−21 %)	2015 (30 %)	−369 (−30 %)	−116 (−21 %)	−52 (−2 %)	−1215 (−30 %)	1751 (28 %)	0	0	0	0	0	0	0	0	0	0	0

*Note*: A-Cr (Crop activities); A-Lv (Livestock activities); A-Ex (Extraction activities); A-Mf (Manufacturing activities); A-Sv (Service activities); C—Cr (Crop commodities); C-Lv (Livestock commodities); C-Ex (Extraction/mining commodities); C-Mf (Manufacturing commodities); C-Sv (Service commodities); L (Labour); K (Capital); hhd (Households); gov (Government); entr (Enterprises), Itax (Indirect taxes); Dtax (Direct taxes), sav-inv (Savings-Investment); row (rest of the world).

*Source*: Authors’ compilation.

Deviations are particularly high for the manufacturing and services accounts (activity and commodity accounts). This pattern reflects reliance on multiple data sources (e.g., the 2017 SAM, the unpublished 2019 SAM, and INSD statistics) to construct intermediate input shares, which increases the likelihood of larger discrepancies in these sectors. Such inconsistencies lead to larger adjustments when the SAM is balanced using the cross-entropy procedure. The observed deviations, therefore, do not indicate structural distortions in the final SAM but reflect (i) the integration of inconsistent data sources and (ii) the relatively large size of these accounts within the intermediate-use matrix, both of which are well-established drivers of higher adjustment magnitudes in the SAM reconciliation procedure.

Compared to the 2019 SAM developed by INSD in 2024, the new SAM overestimates the share of mining and manufacturing in total output by 1 and 4 percentage points, respectively (see Table S10). This is reflected in the value-added and total use shares, which are 7 and 3 percentage points higher for mining, and 5 percentage points higher for manufacturing (see Table S10). Conversely, the output of agriculture and services is up to 3 percentage points lower (see Table S10). The largest differences are in household expenditures for manufacturing and services, with expenditures for services 13 percentage points lower, while expenditures for manufacturing are 14 percentage points higher (see Table S10). The very detailed level of disaggregation we achieved in the new 2019 SAM came at the cost of over- or underestimating some macroeconomic indicators, vis-à-vis the 2019 SAM by INSD. This is a weakness in the SAM development and estimation procedure.

## Limitations

An earlier version of the SAM was presented at a validation workshop at INSD. Feedback and insights gathered during this workshop contributed to refining and finalizing the current version of the SAM. Nevertheless, the SAM continues to face some limitations. First, as explained in the method section, we derived land revenue using an opportunity cost approach. However, the estimation procedure relied on official data sources, including the World Bank, FAO and INSD. Second, we assumed that rural households owned all farmland and received the land income. We base our assumption on the fact that seven of ten plots of farmland in Burkina Faso are exploited and owned by rural households [10]. We employed the share of capital revenue to divide land revenue between poor and non-poor rural households. More valid farmland value and ownership data, which have been unavailable during SAM development, would improve the land account in the SAM. Finally, we aggregated 13 regions into three agroecological zones, which is not entirely accurate, as small parts of some areas belonging to one zone fall under another zone (Fig. 2). Sub-regional data, not available during SAM development, could support a more precise agriculture and household disaggregation by agroecological zone.

## Ethics Statement

The authors have read and follow the ethical requirements for publication in Data in Brief and confirm that the current work does not involve human subjects, animal experiments, or any data collected from social media platforms.

## Credit Author Statement

**Martial A.K. Houessou**: Conceptualization, data collection and development, original draft preparation. **Zuhal Elnour**: Supervision, reviewing, and editing. **Harald Grethe**: Supervision, reviewing, and editing. **François Ramdé**: Data collection, data validation, and reviewing.

**Code Availability**

Robinson and McDonald [3] developed the Cross-Entropy (CE) programme (SAMEST_5) run in GAMS^6^ for the SAM estimation. It is available at https://www.cgemod.org.uk/samest_5.zip. The user guide [11] of the programme is also available to assist users.

## Data Availability

ZENODOA 2019 Social Accounting Matrix for Burkina Faso with Agricultural Activities and Household Groups Disaggregated by Agroecological Zones (Original data).

## Appendix Supplementary materials

Image, application 1

## Footnotes

1 Burkina Faso distinguishes three heterogeneous agroecological zones: the Sudanian (sub-humid), Sudano-Sahelian (semi-arid), and Sahelian (arid) zones [12].

2 XOF, also known as the West African CFA franc (F.CFA), is the currency used by eight West African states, including Benin, Burkina Faso, Côte d’Ivoire, Guinea-Bissau, Mali, Niger, Senegal, and Togo.

3 500,000 XOF is equivalent to 760 Euros.

4 Crops – maize, rice, sorghum, millet & fonio, tuber, peanut, cotton, fruits & vegetables, forestry.

5 Livestock – cattle, pigs, sheep & goats, poultry, fishing and aquaculture.

6 GAMS. General Algebraic Mathematical System (GAMS) software. See the complete documentation of the software on the GAMS website [13].
