# Supplementary material for: A 2019 Social Accounting Matrix for Burkina Faso with Agricultural Activities and Household Groups Disaggregated by Agroecological Zones
Source: Data Brief. 2025 Dec 26;64:112424. doi: 10.1016/j.dib.2025.112424 (PMC12828732; doi:10.1016/j.dib.2025.112424)
Supplement: Supplementary file 1 [file mmc1.docx]

# **Supplementary information**

**Table S1**: Overview of existing SAM for Burkina Faso

| **N°** | **Author, year** | **Title and description** |
| --- | --- | --- |
| 1 | MAHRH, 2016 | *2013 SAM for Burkina Faso*. The SAM comprises 74 activities, 134 products, three types of labour (agricultural, non-agricultural, and family), two types of capital (agricultural capital and non-agricultural), four categories of households (rural-poor, urban-poor, rural-non-poor, and urban-non-poor), three categories of enterprises, government, the rest of the world, and the saving-investment account. |
| 2 | INSD, 2021 | *2015 SAM for Burkina Faso.* The SAM comprises five activities, five products, two factors (labour and capital), five institutions (financial and non-financial enterprises, households, government, and non-profit organisations serving households), two margins (trade and transport), the rest of the world, and the saving-investment account. |
| 3 | INSD, 2021 | *2016 SAM for Burkina Faso.* The SAM comprises five activities, five products, two factors (labour and capital), five institutions (financial and non-financial enterprises, households, government, and non-profit organisations serving households), two margins (trade and transport), the rest of the world, and the saving-investment account. |
| 4 | INSD, 2021 | *2017 SAM for Burkina Faso.* The SAM comprises five activities, five products, two factors (labour and capital), five institutions (financial and non-financial enterprises, households, government, and non-profit organisations serving households), two margins (trade and transport), the rest of the world, and the saving-investment account. |
| 5 | INSD, 2024 | *2019 SAM for Burkina Faso (unpublished).* The SAM comprises 31 activities, 31 products, two factors (labour and capital), two households (poor and non-poor), four other institutions (financial and non-financial enterprises, government, and non-profit organisations serving households), the rest of the world, and the saving-investment account. |

**Table S2**: Accounts in the 2019 Micro-SAM for Burkina Faso (1/3)

| **N°** | **Micro-SAM account** | **Description** |
| --- | --- | --- |
| 1 | a_maize_sd | Maize - Sudanian |
| 2 | a_maize_sh | Maize - Sahelian |
| 3 | a_maize_sdsh | Maize - Sudano-Sahelian |
| 4 | a_rice_sd | Rice - Sudanian |
| 5 | a_rice_sh | Rice - Sahelian |
| 6 | a_rice_sdsh | Rice - Sudano-Sahelian |
| 7 | a_sorgh_sd | Sorghum - Sudanian |
| 8 | a_sorgh_sh | Sorghum - Sahelian |
| 9 | a_sorgh_sdsh | Sorghum - Sudano-Sahelian |
| 10 | a_milfonio_sd | Millet & Fonio - Sudanian |

| 11 | a_milfonio_sh | Millet & Fonio - Sahelian |
| --- | --- | --- |
| 12 | a_milfonio_sdsh | Millet & Fonio - Sudano-Sahelian |
| 13 | a_tuber_sd | Tubers - Sudanian |
| 14 | a_tuber_sh | Tubers - Sahelian |
| 15 | a_tuber_sdsh | Tubers - Sudano-Sahelian |
| 16 | a_cotton_sd | Cotton - Sudanian |
| 17 | a_cotton_sh | Cotton - Sahelian |
| 18 | a_cotton_sdsh | Cotton - Sudano-Sahelian |
| 19 | a_peanut_sd | Peanut - Sudanian |
| 20 | a_peanut_sh | Peanut - Sahelian |
| 21 | a_peanut_sdsh | Peanut - Sudano-Sahelian |
| 22 | a_frtveg_sd | Legumes, Fruits & Vegetables - Sudanian |
| 23 | a_frtveg_sh | Legumes, Fruits & Vegetables - Sahelian |
| 24 | a_frtvegshd | Legumes, Fruits & Vegetables - Sudano-Sahelian |
| 25 | a_cattle_sd | Cattle - Sudanian |
| 26 | a_cattle_sh | Cattle - Sahelian |
| 27 | a_cattle_sdsh | Cattle - Sudano-Sahelian |
| 28 | a_shpgths_sd | Sheep, Goat, Camel, & Horses - Sudanian |
| 29 | a_shpgths_sh | Sheep, Goat, Camel, & Horses - Sahelian |
| 30 | a_shpgths_sdsh | Sheep, Goat, Camel, & Horses - Sudano-Sahelian |
| 31 | a_pig_sd | Pig - Sudanian |

| 32 | a_pig_sh | Pigr- Sahelian |
| --- | --- | --- |
| 33 | a_pigshd | Pig - Sudano-Sahelian |
| 34 | a_poultry_sd | Poultry - Sudanian |
| 35 | a_poultry_sh | Poultry - Sahelian |
| 36 | a_poultry_sdsh | Poultry - Sudano-Sahelian |
| 37 | a_forest_sd | Forestry - Sudanian |
| 38 | a_forest_sh | Forestry - Sahelian |
| 39 | a_forest_sdsh | Forestry - Sudano-Sahelian |
| 40 | a_olivst_sd | Fishing & Aquaculture - Sudanian |
| 41 | a_olivst_sh | Fishing & Aquaculture - Sahelian |
| 42 | a_olivst_sdsh | Fishing & Aquaculture - Sudano-Sahelian |

**Table S2**: Accounts in the 2019 Micro-SAM for Burkina Faso (2/3)

| 43 | a_mining | Mining |
| --- | --- | --- |
| 44 | a_fmanuf | Food Manufacturing |
| 45 | a_omanuf | Non-Food Manufacturing |
| 46 | a_constr | Construction |
| 47 | a_serv | Services |
| 48 | c_maize | Maize |
| 49 | c_rice | Rice |
| 50 | c_sorgh | Sorghum |
| 51 | c_milfonio | Millet & Fonio |
| 52 | c_tuber | Tubers |
| 53 | c_cotton | Cotton |
| 54 | c_peanut | Peanut |
| 55 | c_frtveg | Legumes, Fruits & Vegetables |
| 56 | c_cattle | Cattle |
| 57 | c_shpgths | Sheep, Goat, Camel, & Horses |
| 58 | c_pig | Pig |
| 59 | c_poultry | Poultry |
| 60 | c_forest | Forestry |
| 61 | c_olivst | Fishing & Aquaculture |
| 62 | c_mining | Mining |
| 63 | c_fmanuf | Food Manufacturing |
| 64 | c_omanuf | Non-Food Manufacturing |
| 65 | c_constr | Construction |
| 66 | c_serv | Services |
| 67 | t_mrg | Trade and transport margins |
| 68 | f_unsklab | Unskilled Labour |
| 69 | f_semsklab | Semi-Skilled Labour |
| 70 | f_sklab | Skilled Labour |
| 71 | f_cap | Capital |
| 72 | f_land | Land |
| 73 | h_rurp_sd | Rural Household Poor - Sudanian |
| 74 | h_rurp_sh | Rural Household Poor - Sahelian |
| 75 | h_rurp_sdsh | Rural Household Poor - Sudano-Sahelian |
| 76 | h_urbp_sd | Urban Household Poor - Sudanian |
| 77 | h_urbp_sh | Urban Household Poor - Sahelian |
| 78 | h_urbp_sdsh | Urban Household Poor - Sudano-Sahelian |
| 79 | h_rurnp_sd | Rural Household Non-Poor - Sudanian |
| 80 | h_rurnp_sh | Rural Household Non-Poor -Sahelian |
| 81 | h_rurnp_sdsh | Rural Household Non-Poor - Sudano-Sahelian |
| 82 | h_urbnp_sd | Urban Household Non-Poor - Sudanian |
| 83 | h_urbnp_sh | Urban Household Non-Poor - Sahelian |
| 84 | h_urbnp_sdsh | Urban Household Non-Poor - Sudano-Sahelian |
| 85 | saltax | Sales Taxes |

**Table S2**: Accounts in the 2019 Micro-SAM for Burkina Faso (3/3)

| 86 | Indtax | Production Taxes |
| --- | --- | --- |
| 87 | Dirtax | Direct Taxes |
| 88 | GOVT | Government |
| 89 | ENT | Entreprises |
| 90 | i_s | Savings & Investment |
| 91 | row | Rest of the World |

**Table S3**: List of activity and commodity accounts in the detailed micro-SAM

| **N°** | **Activities** | **N°2** | **Commodities** |
| --- | --- | --- | --- |
| 1 | Maize | 1 | Maize |
| 2 | Rice | 2 | Rice |
| 3 | Sorghum | 3 | Sorghum |
| 4 | Millet & Fonio | 4 | Millet & Fonio |
| 5 | Tubers | 5 | Tubers |
| 6 | Cotton | 6 | Cotton |
| 7 | Peanut | 7 | Peanut |
| 8 | Legumes, Fruits & Vegetables | 8 | Legumes, Fruits & Vegetables |
| 9 | Cattle | 9 | Cattle |
| 10 | Sheep, Goat, Camel, & Horses | 10 | Sheep, Goat, Camel, & Horses |
| 11 | Pig | 11 | Pig |
| 12 | Poultry | 12 | Poultry |
| 13 | Fishing and Aquaculture | 13 | Fishing and Aquaculture |
| 14 | Forestry | 14 | Forestry |
| 15 | Mining | 15 | Mining |
| 16 | Food Manufacturing | 16 | Manufacturing |
| 17 | Non-Food Manufacturing | 16 | Manufacturing |
| 18 | Construction | 17 | Construction |
| 19 | Service | 18 | Service |

**Table S4**: Standards for labour disaggregation

| **N°** | **Standard** | **N°2** | **Sub-standards** |
| --- | --- | --- | --- |
| 1 | Skilled level | 1.1 | Skilled Labour |
|  |  | 1.2 | Semi-skilled labour |
|  |  | 1.3 | Unskilled labour |

**Table S5**: Standards for agriculture disaggregation

| **N°** | **Standard** | **N°2** | **Sub-standards** |
| --- | --- | --- | --- |
| 1 | Agroecological zones | 1.1 | Sudanian |
|  |  | 1.2 | Sudano-Sahelian |
|  |  | 1.3 | Sahelian |

**Table S6**: Standards for household disaggregation

| **N°** | **Standard** | **N°2** | **Sub-standards** |
| --- | --- | --- | --- |
| 1 | Residence place | 1.1 | Rural |
|  |  | 1.2 | Urban |
| 2 | Income | 2.1 | Poor |
|  |  | 2.2 | Non-Poor |
| 3 | Agroecological zones | 3.1 | Sudanian |
|  |  | 3.2 | Sudano-Sahelian |
|  |  | 3.3 | Sahelian |

**Table S7**: Household consumption expenditure in the 2018 HHCL compared to the 2013 SAM and the new 2019 SAM

|  | **2018 HHCL (Base)** | **2013 SAM** | **New 2019 SAM** | **Deviations**  **(2018 HHCL and 2013 SAM)** | **Deviations**  **(2018 HHCL and New 2019 SAM)** |
| --- | --- | --- | --- | --- | --- |
|  | (%) | (%) | (%) | (percentage points) | (percentage points) |
| Food | 47.1 | 59.8 | 52.4 | -12.7 | -5.3 |
| Non-food manufacturing | 32.9 | 22.8 | 26.4 | 10.1 | 6.5 |
| Services | 17.2 | 15.2 | 19.7 | 2.0 | 2.5 |
| Other expenses | 2.8 | 2.2 | 1.5 | 0.6 | 1.3 |
| Total | 100 | 100 | 100 | - | - |

**Note.**

Aggregation based on household expenditure components in the 2013 SAM and new 2019 SAM – **Food** (Maize, Rice, Sorghum, Tubers, Peanut, Legumes, Fruits, Vegetables, Other crops, Cattle, Sheep, Goat, Horse, Pig, Poultry, Other livestock, Food manufacturing); **Non-food manufacturing** (Non-food manufacturing and Construction); **Services** (Services); **Other expenses** (Forestry and Cotton).

Aggregation based on household expenditure components in the 2018 HHCL survey – **Food** (Food, Own-grown food, Food donations); **Non-food manufacturing** (Housing, Durable goods, Clothing and Footwear, Energy, Housing maintenance, and Body care); **Services** (Transport, Health, Education, and Communications); **Other expenses** (Other expenses).

**Source:** Authors’ computation based on MARAH (2016), 2018 HHCL by INSD (2021a), and the new 2019 SAM.

**Table S8**: Production cost structure across aggregate sectors from 2013 to 2019 based on existing SAMs (%)

|  | **Agriculture** | | | | | **Mining/Extraction** | | | | | **Manufacturing** | | | | | **Services** | | | | |
| --- | --- | --- | --- | --- | --- | --- | --- | --- | --- | --- | --- | --- | --- | --- | --- | --- | --- | --- | --- | --- |
|  | 2013 | 2015 | 2016 | 2017 | 2019 | 2013 | 2015 | 2016 | 2017 | 2019 | 2013 | 2015 | 2016 | 2017 | 2019 | 2013 | 2015 | 2016 | 2017 | 2019 |
| Agriculture | 11.0 | 15.2 | 16.1 | 15.3 | 16.0 | 0.0 | 0.1 | 0.1 | 0.0 | 0.0 | 17.3 | 19.0 | 17.7 | 17.3 | 11.7 | 2.8 | 3.1 | 3.2 | 2.8 | 2.7 |
| Mining/Extraction | 0.0 | 0.0 | 0.0 | 0.0 | 0.0 | 1.7 | 0.1 | 0.1 | 1.7 | 3.1 | 0.9 | 0.8 | 1.0 | 0.9 | 1.0 | 0.1 | 0.1 | 0.1 | 0.1 | 0.1 |
| Manufacturing | 7.2 | 14.1 | 14.0 | 13.0 | 14.3 | 30.3 | 37.4 | 33.5 | 30.3 | 29.2 | 37.0 | 34.3 | 34.7 | 37.0 | 27.5 | 14.6 | 15.3 | 14.2 | 14.6 | 15.8 |
| Services | 1.4 | 1.9 | 2.5 | 2.2 | 2.7 | 8.9 | 9.8 | 10.2 | 8.9 | 12.9 | 8.0 | 6.4 | 7.5 | 8.0 | 26.1 | 15.3 | 14.2 | 14.7 | 15.3 | 6.3 |
| Wage compensation | 5.1 | 2.1 | 2.9 | 2.8 | 3.4 | 17.2 | 18.1 | 18.0 | 17.2 | 16.9 | 5.7 | 5.4 | 5.6 | 5.7 | 4.1 | 24.1 | 23.5 | 23.8 | 24.1 | 28.7 |
| Gross operating surplus | 75.4 | 66.8 | 65.3 | 66.8 | 64.1 | 41.2 | 33.4 | 36.9 | 41.2 | 36.7 | 31.3 | 34.0 | 33.8 | 31.3 | 29.8 | 42.7 | 43.4 | 43.5 | 42.7 | 45.8 |
| Production taxes | -0.1 | -0.1 | -0.8 | -0.1 | -0.5 | 0.7 | 1.1 | 1.2 | 0.7 | 1.2 | -0.2 | 0.1 | -0.3 | -0.2 | -0.2 | 0.4 | 0.4 | 0.5 | 0.4 | 0.6 |
| Total | 100 | 100 | 100 | 100 | 100 | 100 | 100 | 100 | 100 | 100 | 100 | 100 | 100 | 100 | 100 | 100 | 100 | 100 | 100 | 100 |

**Table S9**: Change in production cost structure across aggregate sectors from 2013 to 2019 based on existing SAMs (percentage points)

|  | **Agriculture** | | | | | **Mining/Extraction** | | | | | **Manufacturing** | | | | | **Services** | | | | |
| --- | --- | --- | --- | --- | --- | --- | --- | --- | --- | --- | --- | --- | --- | --- | --- | --- | --- | --- | --- | --- |
|  | 2013  (Base) | 2015 | 2016 | 2017 | **2019** | 2013  (Base) | 2015 | 2016 | 2017 | **2019** | 2013  (Base) | 2015 | 2016 | 2017 | **2019** | 2013  (Base) | 2015 | 2016 | 2017 | **2019** |
| Agriculture | 0.0 | 4.2 | 5.1 | 4.3 | **5.0** | 0.0 | 0.1 | 0.1 | 0.0 | **0.0** | 0.0 | 1.7 | 0.4 | 0.0 | **-5.6** | 0.0 | 0.3 | 0.4 | 0.0 | **0.1** |
| Mining/Extraction | 0.0 | 0.0 | 0.0 | 0.0 | **0.0** | 0.0 | -1.6 | -1.5 | 0.0 | **1.4** | 0.0 | -0.1 | 0.1 | 0.0 | **0.1** | 0.0 | 0.0 | 0.0 | 0.0 | **0.0** |
| Manufacturing | 0.0 | 6.8 | 6.8 | 5.8 | **7.1** | 0.0 | 7.2 | 3.2 | 0.0 | **-1.2** | 0.0 | -2.8 | -2.4 | 0.0 | **-9.6** | 0.0 | 0.7 | -0.5 | 0.0 | **1.2** |
| Services | 0.0 | 0.6 | 1.1 | 0.8 | **1.4** | 0.0 | 0.9 | 1.3 | 0.0 | **4.1** | 0.0 | -1.6 | -0.5 | 0.0 | **18.1** | 0.0 | -1.1 | -0.6 | 0.0 | **-8.9** |
| Wage compensation | 0.0 | -3.0 | -2.2 | -2.3 | **-1.7** | 0.0 | 0.9 | 0.8 | 0.0 | **-0.3** | 0.0 | -0.3 | -0.1 | 0.0 | **-1.6** | 0.0 | -0.6 | -0.3 | 0.0 | **4.6** |
| Gross operating surplus | 0.0 | -8.6 | -10.1 | -8.6 | **-11.2** | 0.0 | -7.8 | -4.3 | 0.0 | **-4.5** | 0.0 | 2.7 | 2.5 | 0.0 | **-1.5** | 0.0 | 0.7 | 0.8 | 0.0 | **3.1** |
| Production taxes | 0.0 | 0.0 | -0.7 | 0.0 | **-0.4** | 0.0 | 0.4 | 0.6 | 0.0 | **0.5** | 0.0 | 0.3 | -0.1 | 0.0 | **0.0** | 0.0 | 0.0 | 0.1 | 0.0 | **0.3** |

**Note**. Wage compensation accounts for only hired labour, while the gross operating surplus (also referred to as income mixed) accounts for family labour, land, and actual capital.

**Source**: Authors' computation based on MAHRH (2016), INSD (2021) and Unpublished SAM by INSD (2024).

**Table S10**: Comparisons of key macroeconomic indicators between the newly developed 2019 SAM and the INSD 2019 SAM

|  | **Sectors** | **New SAM 2019** | **INSD 2019 SAM** | **Deviations** |
| --- | --- | --- | --- | --- |
|  |  | (%) | (%) | (percentage points) |
| Shares in total intermediate consumption | Agriculture | 19.2 | 18.3 | 0.9 |
|  | Mining | 8.4 | 2.0 | 6.4 |
|  | Manufacturing | 55.5 | 54.1 | 1.4 |
|  | Services | 17.0 | 25.6 | -8.6 |
|  | **Total** | **100** | **100** |  |
| Shares in value-added | Agriculture | 14.1 | 19.7 | -5.6 |
|  | Mining | 19.6 | 12.8 | 6.8 |
|  | Manufacturing | 22.6 | 17.9 | 4.6 |
|  | Services | 43.7 | 49.6 | -5.8 |
|  | **Total** | **100** | **100** |  |
| Output shares | Agriculture | 16.0 | 19.0 | -3.0 |
|  | Mining | 15.3 | 14.3 | 1.0 |
|  | Manufacturing | 35.5 | 31.8 | 3.7 |
|  | Services | 33.2 | 34.9 | -1.7 |
|  | **Total** | **100** | **100** |  |
| Supply shares | Agriculture | 14.9 | 16.4 | -1.4 |
|  | Mining | 1.2 | 1.9 | -0.7 |
|  | Manufacturing | 52.3 | 46.4 | 5.8 |
|  | Services | 31.6 | 35.3 | -3.7 |
|  | **Total** | **100** | **100** |  |
| Shares in total use | Agriculture | 14.0 | 15.5 | -1.5 |
|  | Mining | 14.4 | 11.0 | 3.4 |
|  | Manufacturing | 47.5 | 42.8 | 4.7 |
|  | Services | 24.1 | 30.7 | -6.6 |
|  | **Total** | **100** | **100** |  |
| Household income shares | Factor | 93.7 | 90.2 | 3.5 |
|  | Government | 2.5 | 2.9 | -0.4 |
|  | Remittances | 1.8 | 3.8 | -2.1 |
|  | Enterprises | 2.1 | 3.1 | -1.0 |
|  | **Total** | **100** | **100** |  |
| Household expenditure shares | Agriculture | 19.9 | 20.3 | -0.5 |
|  | Mining | 0.0 | 0.0 | 0.0 |
|  | Manufacturing | 60.5 | 47.0 | 13.5 |
|  | Services | 19.7 | 32.7 | -13.1 |
|  | **Total** | **100** | **100** |  |

**Source**: Authors' computation
